# Characterization of Recombinant Adeno-Associated Viruses (rAAVs) for Gene Therapy Using Orthogonal Techniques

**DOI:** 10.3390/pharmaceutics13040586

**Published:** 2021-04-20

**Authors:** Liam Cole, Diogo Fernandes, Maryam T. Hussain, Michael Kaszuba, John Stenson, Natalia Markova

**Affiliations:** Malvern Panalytical Ltd., Enigma Business Park, Grovewood Road, Malvern, Worcestershire WR14 1XZ, UK; liam.cole@malvernpanalytical.com (L.C.); diogo.fernandes@malvernpanalytical.com (D.F.); maryam.hussain@malvernpanalytical.com (M.T.H.); john.stenson@malvernpanalytical.com (J.S.); natalia.markova@malvernpanalytical.com (N.M.)

**Keywords:** recombinant adeno-associated viruses (rAAV), dynamic light scattering (DLS), multiangle dynamic light scattering (MADLS^®^), high-throughput, orthogonal techniques, SEC–MALS, differential scanning calorimetry (DSC)

## Abstract

Viruses are increasingly used as vectors for delivery of genetic material for gene therapy and vaccine applications. Recombinant adeno-associated viruses (rAAVs) are a class of viral vector that is being investigated intensively in the development of gene therapies. To develop efficient rAAV therapies produced through controlled and economical manufacturing processes, multiple challenges need to be addressed starting from viral capsid design through identification of optimal process and formulation conditions to comprehensive quality control. Addressing these challenges requires fit-for-purpose analytics for extensive characterization of rAAV samples including measurements of capsid or particle titer, percentage of full rAAV particles, particle size, aggregate formation, thermal stability, genome release, and capsid charge, all of which may impact critical quality attributes of the final product. Importantly, there is a need for rapid analytical solutions not relying on the use of dedicated reagents and costly reference standards. In this study, we evaluate the capabilities of dynamic light scattering, multiangle dynamic light scattering, and SEC–MALS for analyses of rAAV5 samples in a broad range of viral concentrations (titers) at different levels of genome loading, sample heterogeneity, and sample conditions. The study shows that DLS and MADLS^®^ can be used to determine the size of full and empty rAAV5 (27 ± 0.3 and 33 ± 0.4 nm, respectively). A linear range for rAAV5 size and titer determination with MADLS was established to be 4.4 × 10^11^–8.7 × 10^13^ cp/mL for the nominally full rAAV5 samples and 3.4 × 10^11^–7 × 10^13^ cp/mL for the nominally empty rAAV5 samples with 3–8% and 10–37% CV for the full and empty rAAV5 samples, respectively. The structural stability and viral load release were also inferred from a combination of DLS, SEC–MALS, and DSC. The structural characteristics of the rAAV5 start to change from 40 °C onward, with increasing aggregation observed. With this study, we explored and demonstrated the applicability and value of orthogonal and complementary label-free technologies for enhanced serotype-independent characterization of key properties and stability profiles of rAAV5 samples.

## 1. Introduction

Viruses and their derivatives have many applications in biomedicine, biotechnology, and nanotechnology [1,2,3,4]. In gene therapy, viruses are used as vectors to deliver modified genes into target cells, to treat or prevent a disease by replacing a mutated gene that causes disease with a healthy copy of the gene, inactivating or ‘knocking out’ a mutated gene that is functioning improperly, or introducing a new gene into the body to help fight a disease or treat a syndrome [5,6,7,8,9,10,11].

Adenoviruses, retroviruses, lentiviruses, adeno-associated viruses (AAVs), and other human and animal viruses have been utilized for the development of gene therapies over several decades [12]. In recent years, this application area has seen significant growth, largely due to developments in recombinant AAV vector engineering and production for in vivo gene therapies [13].

Recombinant adeno-associated viruses (rAAV) are a class of viral vectors that are being investigated intensively in the development of gene therapies [14,15]. In order to develop efficient rAAV therapies, produced through controlled and economical manufacturing processes, multiple challenges need to be addressed, from capsid design through identification of optimal process and formulation conditions to comprehensive quality control of drug substance and drug product [16,17].

These growing requirements warrant fit-for-purpose analytics and extended characterization through orthogonal use of multiple technologies. Accurate quantification and characterization of rAAVs can accelerate the development and advancement of gene therapies. Addressing these challenges requires extensive characterization of rAAV samples with multiple assays including measurements of capsid count, full–empty capsid ratio, particle size, aggregate formation, stability, genome release, and capsid charge [18]. Typically, rAAV titer and viral load are measured using a combination of enzyme-linked immunosorbent assay (ELISA) [19,20], quantitative polymerase chain reaction (qPCR) [21], droplet digital polymerase chain reaction (ddPCR) [22,23], analytical ultracentrifugation (AUC) [24], and electron microscopy (EM) techniques. These methods are generally time-consuming and labor-intensive, and they have questionable accuracy and precision [24]. Thus, new technologies need to be investigated.

Technologies such as dynamic light scattering (DLS), multiangle dynamic light scattering (MADLS), electrophoretic light scattering (ELS), size-exclusion chromatography multiangle light scattering (SEC–MALS), nanoparticle tracking analysis (NTA), isothermal titration calorimetry (ITC), and differential scanning calorimetry (DSC) can provide important information on the key analytical and quality attributes of viral vectors, enabling the characterization, comparison, and optimization of various parameters. Table 1 summarizes various critical quality attributes (CQA) that are important in viral vector research, together with the techniques that can provide such information, and these are covered in this study [17].

DLS, MADLS, SEC–MALS, NTA, ITC, and DSC are label-free biophysical techniques which require minimal assay development and can be readily applied at all stages, strengthening the analytical workflow for gene therapy development. In this study, recombinant adeno-associated viruses were characterized to achieve convergence of evidence from multiple techniques. For the purposes of the reader, the techniques used in this work are summarized here. Further information can be found in the references listed for each technique.

Dynamic light scattering (DLS) is a noninvasive technique that measures the time-dependent fluctuations in the scattering intensity arising from a particle dispersion or molecular solution. The intensity of the scattered light fluctuates due to the random movement of the particles or molecule undergoing Brownian motion. Analysis of these intensity fluctuations using auto correlation determines the translational diffusion coefficients and, subsequently, the hydrodynamic size using the Stokes–Einstein relationship [25,26,27,28,29].

Multiangle dynamic light scattering (MADLS) allows for a higher-resolution size determination of multimodal samples, by using three different detection angles (back, side, and forward) and combining the information obtained into one angle-independent particle size distribution. An extension of MADLS is particle concentration measurements giving the total particle concentration and the particle concentration for each mode present in a sample [30,31,32,33].

Electrophoretic light scattering (ELS) measures the frequency shift of scattered light from particles or molecules undergoing electrophoresis and enables the measurement of zeta potential. The zeta potential of a particle is the overall charge that the particle acquires in a particular medium, and it can be used to predict the stability of the dispersion and provide insights into the surface chemistry of the particle being investigated [34,35,36,37].

Size-exclusion chromatography (SEC) is a technique that separates molecules according to their hydrodynamic radius as they enter and exit the pores of a porous gel packing matrix in a column. A range of advanced detectors, such as light scattering, UV, RI, and viscosity, allows for the measurement of absolute molecular weight, molecular size, intrinsic viscosity, branching, and other parameters [38].

Differential scanning calorimetry (DSC) is an analysis technique used to characterize the stability of a protein or other biomolecule directly in its native form without the need for extrinsic or intrinsic fluorophores. It does this by measuring the heat capacity change associated with thermally induced processes, such as the molecule’s thermal denaturation, triggered by heating at a constant rate [39,40].

This paper discusses and demonstrates the applicability and value of various orthogonal and complementary label-free technologies for enhanced serotype-independent characterization of key properties and stability profiles of rAAV5 samples.

## 2. Materials and Methods

### 2.1. Materials

Commercial rAAV samples (rAAV5) were purchased as nominally empty and nominally full rAAV5 from Virovek (Hayward, CA, USA). From information provided by the supplier, the rAAV vectors were purified through two rounds of CsCl-gradient ultracentrifugation followed by sterile filtration of the nominally empty and nominally full fractions. The rAAV samples were used as received following the manufacturer’s specifications for purity and titer. These AAV capsids are referred to as empty and full throughout the rest of the paper. The full rAAV5 contained pFB-GFP ssDNA which consisted of 2544 nucleotides with a known molecular weight (Mw) of 785,000 g/mol. The sample had a defined viral titer of 2.5 × 10^13^ viral genome per mL (vg/mL) as calculated by qPCR. The disperse phase of the samples was PBS containing 0.001% Pluronic F-68 (Virovek, Hayward, CA, USA). The 100 nm polystyrene latex sizing standards (part number 3100A) were obtained from Thermo Scientific (Waltham, MA, USA). Milli-Q^®^ filtered water was obtained from Milli-Q Direct Water Purification System (Merck Millipore, Livingston UK). 

### 2.2. Size-Exclusion Chromatography Multiangle Light Scattering (SEC–MALS)

Chromatographic separation of the rAAV samples was achieved using a Superose 6 increase (10/300) with an isocratic flow rate of 0.8 mL/min and phosphate-buffered saline as the mobile phase. An OMNISEC system (Malvern Panalytical Ltd., Malvern, UK) consisting of an OMNISEC RESOLVE (pump, autosampler, and column oven) and an OMNISEC REVEAL (refractive index, UV/Vis-PDA and right-angle/low-angle light scattering detector) was used to acquire the sample chromatograms. The samples were maintained at 4 °C in the autosampler prior to injection. The column oven and detector module were maintained at a constant 30 °C during this work.

Following separation, the samples were processed using a compositional analysis designed to determine the concentration and molecular weight of two distinct components within a sample. For the compositional analysis to work, it is necessary to know the refractive index increment (dn/dc) and extinction coefficient (dA/dc) of both components. In this case, the dn/dc of the capsid and the ssDNA is well known. The dA/dc for the capsid can be measured using OMNISEC, and the dA/dc for the ssDNA is calculated from the sequence.

Total particle (Equation (1)), full particle (Equation (2)), and empty particle (Equation (3)) concentration can all be obtained using the following equations:

C_total_ = (Conc_Capsid_ × N_A_)⁄(Mw_Capsid_), (1)

C_full_ = (Conc_DNA_ × N_A_)⁄(Mw_Seq DNA_), (2)

C_Empty_ = C_total_ − C_full_, (3)

where Conc_Capsid_ is the concentration of the capsid in mg/mL as calculated, N_A_ is Avogadro’s number, Mw_capsid_ is the molecular weight (g/mol) of the capsid as calculated, Conc_DNA_ is the concentration of the DNA in mg/mL as calculated, and Mw_Seq DNA_ is the molecular weight of the ssDNA from the sequence. Therefore, using these calculated particle concentrations, the percentage of full rAAV5 in a sample can be easily derived. The compositional analysis of SEC–MALS data in this study is based on a simplified model where capsids are treated as empty or filled only.

### 2.3. Dynamic Light Scattering (DLS)

Dynamic light scattering (DLS) thermal ramp measurements were made with a Zetasizer Ultra (Malvern Panalytical Ltd., Malvern, UK) using an He–Ne laser at a wavelength of 633 nm and maximum power of 10 mW. Three repeat measurements of each sample were made at each temperature using backscatter detection and a low-volume quartz batch cuvette (ZEN2112 Malvern Panalytical Ltd., Malvern, UK). The instrument settings were optimized automatically by means of the ZS XPLORER software (Malvern Panalytical Ltd., Malvern, UK).

The thermal ramps covered a temperature range of 45 to 75 °C with 1 °C increments, with size measurements collected at every 1 °C increase and a particle concentration measurement taken every 5 °C.

### 2.4. Multiangle Dynamic Light Scattering (MADLS)

Multiangle dynamic light scattering (MADLS) particle concentration measurements were carried out using a Zetasizer Ultra (Malvern Panalytical Ltd., Malvern, UK) equipped with an He–Ne laser at a wavelength of 633 nm and maximum power of 10 mW. All experiments were performed at 25 °C using a sample volume of 20 µL and a low-volume quartz batch cuvette (ZEN2112 Malvern Panalytical Ltd., Malvern, UK). The instrument settings were optimized automatically by means of the ZS XPLORER software (Malvern Panalytical Ltd., Malvern, UK). A series of sample dilutions in PBS with 0.001% Pluronic F-68 buffer were prepared gravimetrically and measured to determine viral titer linearity. Each result reported was the average of 10 repeat measurements.

Determination of dispersant viscosity was made by doping a small aliquot (2 µL) of a 100 nm polystyrene latex sizing standard (Thermo Scientific, Waltham, MA, USA) into 1 mL of each sample, assuming the viscosity of water and back calculating the correct viscosity to obtain the correct size for the polystyrene latex sizing standard [41,42]. The dispersant viscosity was determined to be 0.98 mPa·s at 25 °C, and a dispersant refractive index of 1.331 was used for the MADLS and ELS measurements.

The optical properties used for the measurements were derived empirically through orthogonal light scattering technology. The values used in this study were particle refractive indices of 1.45 (empty rAAVs) and 1.50 (full rAAVs) with an absorption of 0.001. The scattering intensity of the dispersant was measured to be 68 kilocounts per second (kcps) and used during particle concentration measurements.

### 2.5. Electrophoretic Light Scattering (ELS)

Electrophoretic light scattering (ELS) measurements of the rAAV samples were measured on a Zetasizer Ultra instrument (Malvern Panalytical Ltd., Malvern, UK) equipped with an He–Ne laser at a wavelength of 633 nm and maximum power of 10 mW. Measurements were performed using the diffusion barrier method (DBM) [37,43,44,45,46] in which 20 µL aliquots of the samples were introduced into a folded capillary cell (DTS1070 Malvern Panalytical Ltd., Malvern, UK) containing the PBS with 0.001% Pluronic F-68 buffer. The instrument settings were optimized automatically by means of the ZS XPLORER software. All measurements were made at 25 °C and consisted of (1) five repeat DLS size measurements using back scatter detection, (2) five repeat ELS zeta potential measurements with a 60 s delay between each measurement to minimize Joule heating and polarization effects, and (3) five repeat DLS size measurements using backscatter detection. The use of DLS size measurements before and after the ELS measurements was to confirm that the application of a voltage did not cause the integrity of the samples to be compromised. The field strength used was approximately 8 V/cm, and the measured electrophoretic mobilities were converted into zeta potentials using the Smoluchowski approximation [34,35,36].

### 2.6. Differential Scanning Calorimetry (DSC)

MicroCal PEAQ DSC automated (Malvern Panalytical, Northampton, MA, USA) was used for analysis of the thermal stability of empty and full rAAV5 samples. For the analysis, rAAV5 samples and matching buffer solutions at 325 μL aliquots were loaded onto a 96-well plate, covered with a silicon seal, and placed into the PEAQ DSC plate stacker thermostatically regulated at 10 °C. The empty and full rAAV5 samples were used at concentration of 3.6 × 10^13^ particles/mL and 4.3 × 10^13^ particles/mL, respectively. Mean molecular weight of the rAAV5 viral capsid protein was estimated on the basis of the reported 1:1:10 molar ratio for VP1:VP2:VP3 capsid proteins to be 64,900 g/mol. The protein monomer-based molar concentrations of the empty and full rAAV5 samples were established as 3.56 and 4.3 μM, respectively [47].

The thermal scans were performed in the range from 20 °C to 110 °C at a scan rate of 60 °C/h. In between the sample measurements, the sample and the reference cells of the PEAQ DSC instrument were automatically cleaned with 10% *v/v* Decon 90 solutions following a SCAN cleaning procedure, replicating the scanning conditions used in the measurements and including a thorough rinse with Milli-Q^®^ filtered water.

The data were analyzed with dedicated PEAQ DSC Analysis software (Malvern Panalytical, Northampton, MA, USA). For analysis, sample thermograms were normalized for the sample concentration and corrected for the instrument baseline by subtraction of the corresponding buffer–buffer scan. Lastly, the sample thermograms were automatically baseline-corrected following extrapolation of pre- and post-transition baselines with a spline function. The resulting normalized and baseline-corrected DSC traces of the full and empty rAAV5 samples were analyzed for T_onset_, T_m_ and enthalpy of transition, Δ*H*_main tr_.

## 3. Results

### 3.1. SEC–MALS

**Figure 1:**
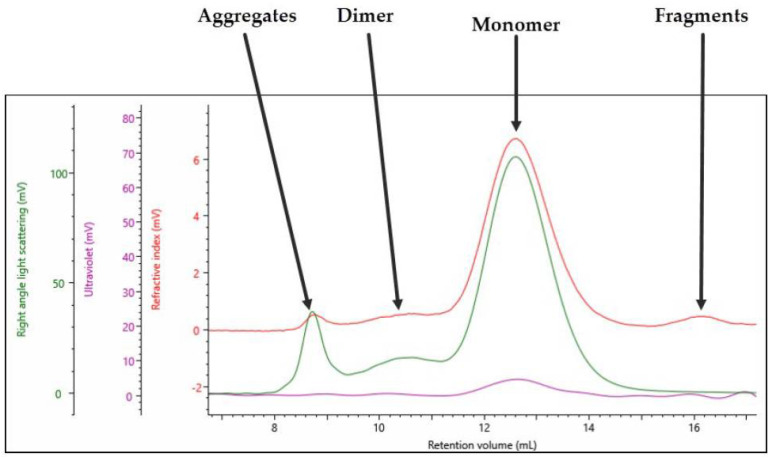
Triple detection chromatogram of rAAV5 (Empty). Red = RI; purple = UV 260 nm; green = RALS.

The multi-detection chromatogram for empty rAAV5 is shown in Figure 1. The RI signal is represented by the red channel, the UV at 260 nm is represented by the purple channel, and the right-angle light scattering (RALS) detector is represented by the green channel.

As labeled in Figure 1, the sample contains four populations: the main monomer peak at 12.5 mL retention volume (Rv), fragments at 16 mL Rv, the dimer at 10.5 mL Rv, and aggregates toward the void volume of the column at 8.5 mL Rv. From the SEC–MALS results shown in Section 3.1, a compositional analysis method discussed in Section 2.2 was used to generate the data in Table 2 for the empty rAAVs.

**Figure 2:**
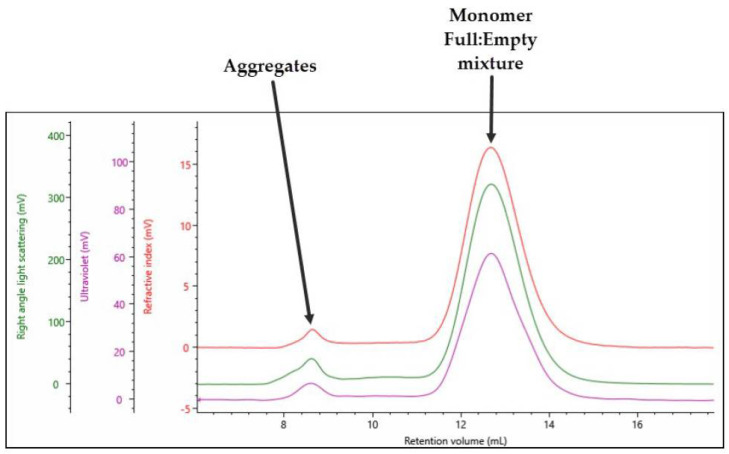
Triple detection chromatogram of rAAV5 (Full). Red = RI; purple = UV 260 nm; green = RALS.

The triple detection chromatogram for full rAAV5 is shown in Figure 2. A significantly different profile to that observed with the empty rAAV5 is shown. In this case, there are only two distinct populations: the monomer peak, which is expected to contain a mixture of full and empty rAAV5 at 12.5 mL Rv, and an aggregate peak at 8 mL Rv.

Using the compositional analysis method, the data in Table 3 can be generated for the full rAAVs.

**Figure 3:**
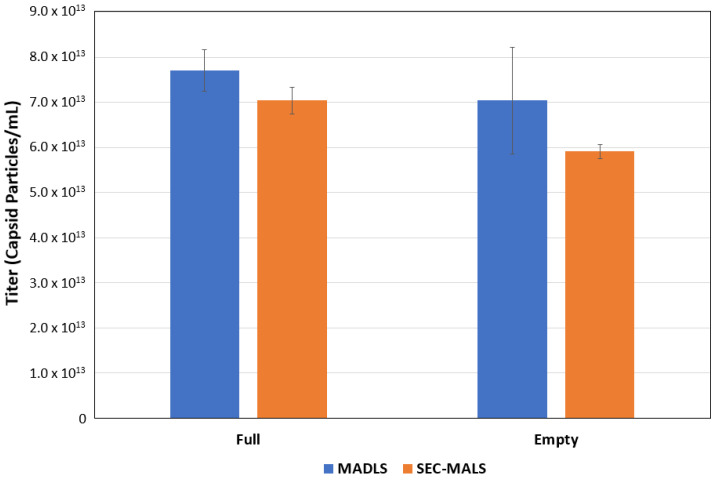
Comparison of full and empty rAAV titer (capsid particles/mL) using SEC–MALS and MADLS.

The data obtained from OMNISEC can be compared with the particle titer from the Zetasizer Ultra (Figure 3) and show good correlation between these two orthogonal techniques.

The key CQA parameter which can be calculated by OMNISEC is the percentage of full rAAV5 in the sample. In this case, the monomer was found to be 78% full rAAV5 with 22% empty rAAV5. Importantly, this analysis method assumes that the sample is either full or empty, whereas it is not possible to account for partly filled or overfilled particles.

**Figure 4:**
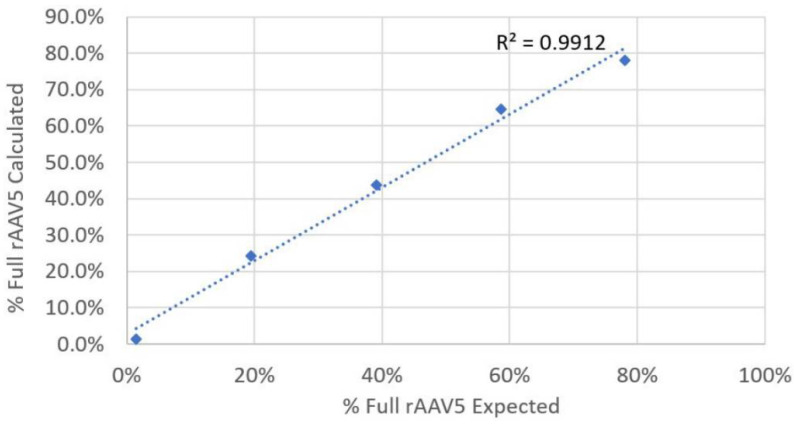
A plot of the expected and calculated percentage of full rAAV5 virus particles.

To confirm the analysis of full rAAV5 samples, the previously analyzed full and empty samples were mixed in controlled ratios. Table 4 shows the expected and the calculated percentage of full AAV per sample. Figure 4 shows the plot of expected and calculated percentage of full rAAV5. This study was used as a proof of concept, and the data presented in Table 4 and Figure 4 are derived from single SEC–MALS measurements. As can be seen in Figure 3, a strong correlation between the expected and the calculated values was obtained, confirming the reliability of OMNISEC to determine this CQA.

The capabilities of OMNISEC for detailed characterization of rAAV samples were further evaluated in the context of a limited stability study, where the rAAV5 sample was subjected to thermal ramps between 25 °C and 80 °C using the Zetasizer Ultra in backscatter detection, which has high sensitivity to minor changes in the aggregation profile of the sample.

**Figure 5:**
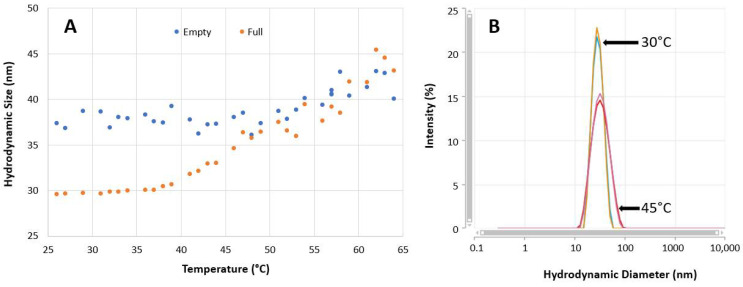
Thermal stress conditions for full and empty rAAV5 stability study with the Zetasizer Ultra. (**A**) Hydrodynamic diameter trend with temperature. (**B**) Overlay of intensity particle size distributions at 30 °C and 45 °C for full rAAV5 samples.

The hydrodynamic sizes [25,26,27,28] of full rAAV5 sample were measured at each temperature, and the results are summarized in Figure 5A. Between 25 °C and 35 °C, no change in hydrodynamic size was observed. An increase in size was recorded from 35 °C, suggesting sample change. Figure 5B shows the intensity particle size distributions obtained from MADLS measurements at 30 °C and 45 °C for the full rAAV5 capsids and clearly shows the differences between the samples.

**Figure 6:**
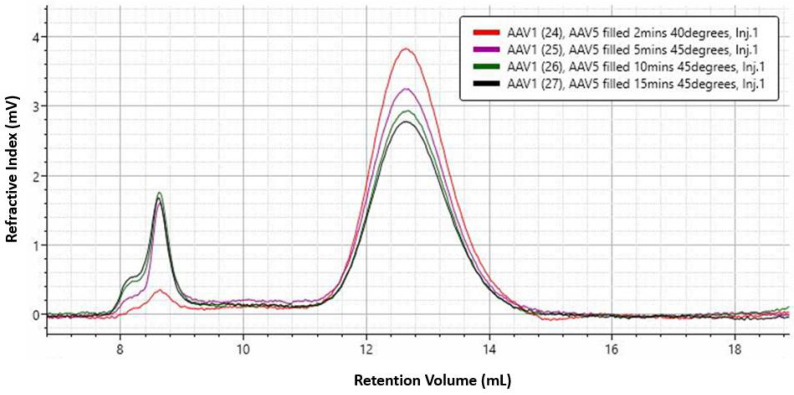
RI detection chromatograms of full rAAV5 samples incubated at 45 °C and aliquoted at 2, 5, 10, and 15 min for analysis by SEC–MALS.

The 45 °C condition was selected for a limited stress stability study, followed by high-resolution multiple-detection SEC. rAAV5 samples were incubated at 45 °C, and aliquots were taken at 2, 5, 10, and 15 min for measurement by SEC–MALS. The chromatograms (Figure 6) show a clear change in the sample with increasing aggregation and decreasing monomer concentration. Table 5 shows that the complex molecular weight remains stable during this incubation period, as does the percentage of full AAV in the monomer peak. This suggests that neither population, empty or full, is being preferentially aggregated.

### 3.2. DLS

**Figure 7:**
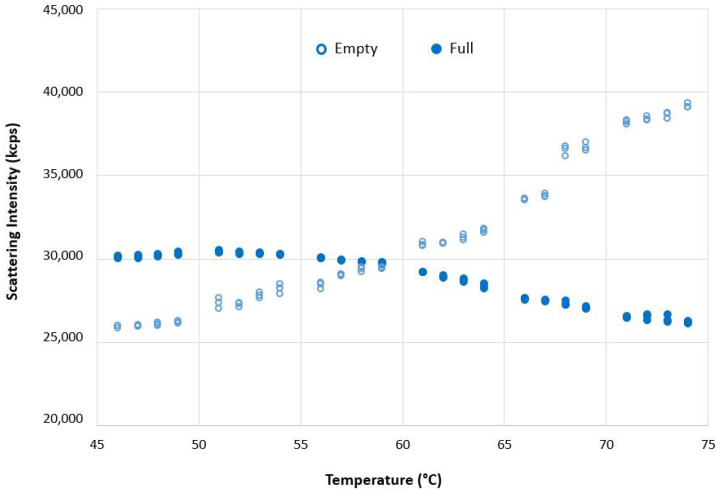
A plot of the scattering intensities (in kilocounts per second (kcps)) as a function of temperature for full and empty rAAV5 samples.

Figure 7 shows a plot of the scattering intensities (in kilocounts per second (kcps) as a function of temperature for full and empty rAAV5 samples obtained from DLS thermal ramp measurements.

**Figure 8:**
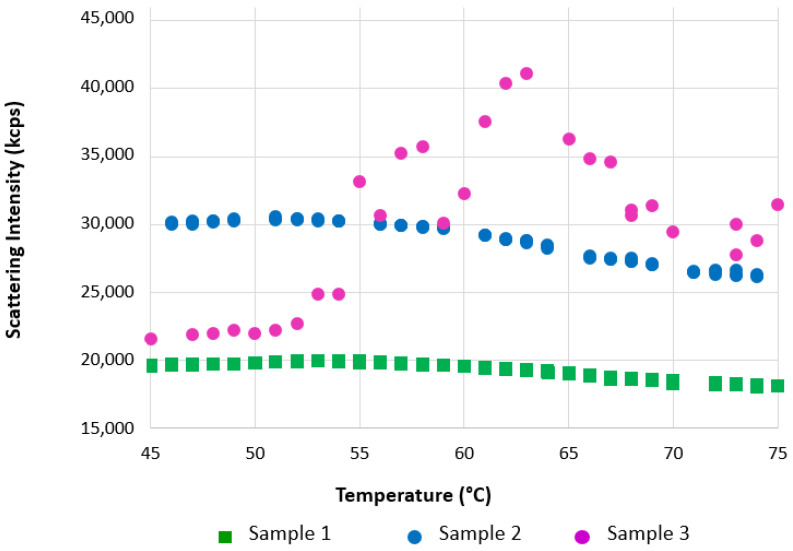
Thermal ramps of full rAAV5 capsids using different buffer and experimental conditions. Sample 1 = PBS at 1 °C/min; Sample 2 = PBS at 3 °C/min; Sample 3 = PBS plus 20 mM EDTA at 1 °C/min.

Figure 8 summarizes the results of thermal ramps of full rAAV5 capsids using different buffer and experimental conditions.

### 3.3. MADLS

**Figure 9:**
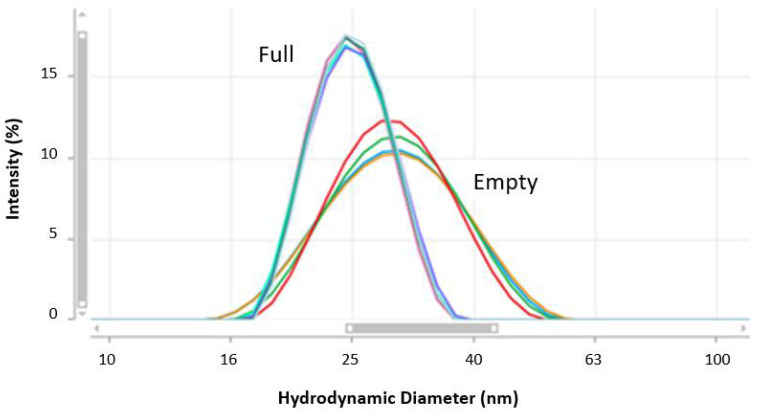
Intensity particle size distributions obtained for empty and full rAAV samples using MADLS.

The results obtained from repeat MADLS measurements of neat concentrations of empty and full rAAV5 samples are shown in Figure 9 and summarized in Table 6. Differences in the hydrodynamic diameters and percentage polydispersity values of the two samples were determined, with full rAAV5 having a hydrodynamic size of 25 nm and percentage polydispersity of 0.2 compared to a size of 32 nm and polydispersity of 0.36 for empty rAAV5.

**Figure 10:**
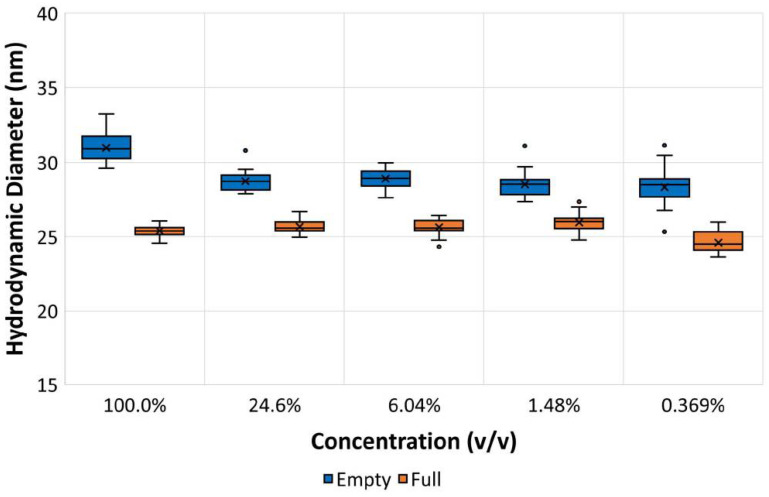
Hydrodynamic sizes of full and empty rAAV5 samples measured at a range of dilutions expressed in volume-by-volume percentages using MADLS measurements.

To mimic viral vector titers typically seen during development, MADLS size measurements were conducted on a broad range of dilutions for both full and empty rAAV5 samples. The results are summarized in Figure 10, which is a boxplot presenting the descriptive statistics of a large dataset including points defined by the analysis as outliers. The data show excellent consistency of size measurement for empty and full rAAV5 samples across a log_2_ dilution range.

**Figure 11:**
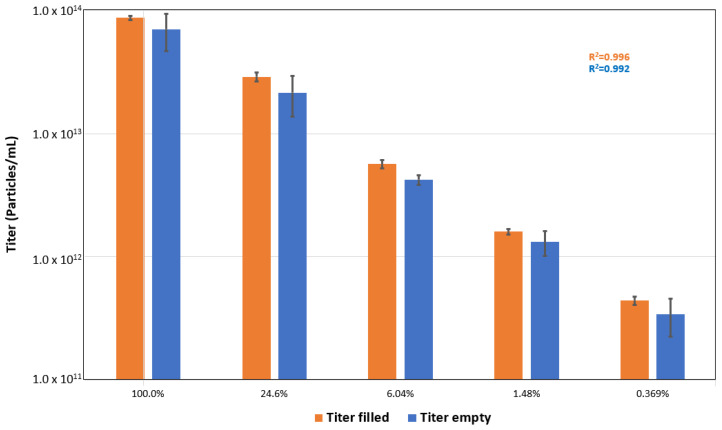
Particle concentrations values obtained for empty and full rAAV5 samples across a range of dilutions expressed in volume-by-volume percentages.

The results of MALDS particle concentration measurements for various dilutions of the empty and full rAAV samples are summarized in Figure 11. The data for both sample types show excellent linearity across the dilution range studied with *R*^2^ values of 0.992 and 0.996, respectively.

The average titer values for the neat concentration (100%) filled and empty rAAV5 samples (8.7 × 10^13^ and 7.0 × 10^13^ particles/mL respectively) were consistent with the concentrations reported by Virovek (Section 2.1).

### 3.4. ELS

The results from ELS measurements of empty and full rAAV5 samples are summarized in Table 7. The data show good repeatability and a significant difference in the electrophoretic mobilities (and, hence, zeta potential values) of the two samples with the full rAAV5 samples measuring −17.7 mV (±1.9 mV) compared to −7.7 mV (±2.2 mV) for the empty rAAV5. DLS backscatter sizing measurements performed before and after the ELS measurements showed consistent particle sizes for both samples (data not shown), confirming that the integrity of the capsids was maintained.

### 3.5. DSC

The results on the thermal stability of rAAV5 samples are summarized in Table 8. In agreement with the published data on the AAV thermal stability, T_m_ values of main transitions for the empty and full rAAV5 samples are similar and characteristic of AAV5 serotype [48]. Content of the folded rAAV5 capsid proteins undergoing main transition in the nominally full and nominally empty rAAV5 samples was similar, according to the transition enthalpy of the main peaks, and it corroborated capsid titer values determined by MADLS and OMNISEC.

**Figure 12:**
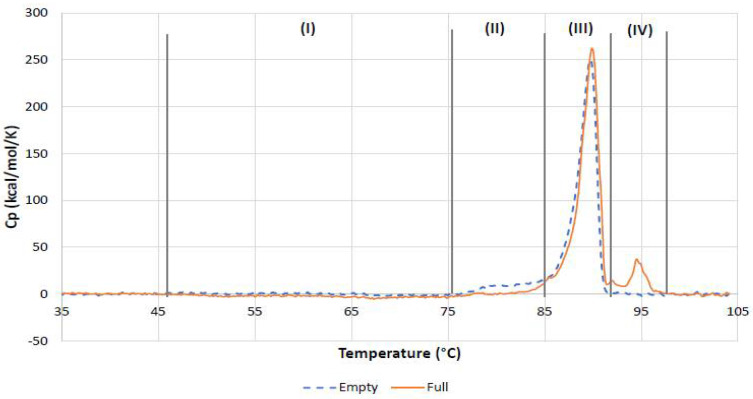
Overlay of the DSC traces of nominally empty and nominally full rAAV5 samples. DSC data corrected for the instrumental blank and the baseline. Vertical lines mark regions with distinctly different regimes of thermally induced processes.

However, several differences can be identified (Figure 12) such as (a) significantly different Tonset values, (b) a pretransition deep on the DSC trace of the full rAAV5 sample, (c) a broad pretransition shoulder on the DSC trace of the empty AAV5, and (d) a well-resolved additional transition only detected for the full AAV5 sample.

## 4. Discussion

The key piece of data generated from SEC–MALS measurements is the absolute molecular weight, independent of column retention volume or any standards used to calibrate the system. In the case of empty rAAVs (Figure 1 and Table 2), the Mw of the main monomer is 3.84 × 10^6^ g/mol. The theoretical molecular weight of the empty capsid is 3.8 × 10^6^ g/mol, confirming that this analysis is working as expected.

The Mw/Mn describes the dispersity of a sample, with a value close to 1 suggesting a single population within a peak and a value much higher than 1 suggesting multiple populations within a peak. In the case of empty rAAVs, the monomer and dimer have values close to 1, suggesting single populations. The aggregates and fragments are significantly higher than 1, suggesting multiple populations with different molecular weights within the single peak (Table 2).

The fraction of the sample describes how the sample is distributed between populations and, in this case, 84.7% of the sample is a monomer. The fraction of protein shows the percentage of capsid within the sample; in this case, the monomer is 99.8% capsid. This confirms that the sample is empty rAAV5. The recoding of 0.2% DNA within the sample is probably due to the protein having minor absorbance at 260 nm and can be taken as a source of error in any future %AAV-filled calculations. The final critical piece of information obtained from this single analysis method is the sample titer; in this case, for the empty rAAV5, a titer of 5.91 × 10^13^ vp/mL was measured.

The SEC–MALS analysis of full rAAV5 capsids was shown in Figure 2 and the data were summarized in Table 3. For the main monomer peak, a complex Mw of 4.49 × 10^6^ g/mol was calculated of which 86% was measured to be capsid. This gives the protein component of the filled rAAV5 an Mw of 3.89 × 10^6^ g/mol, which is in line with the data generated for the empty rAAV5 capsids in Table 2. The monomer is 93% of the total population, and the sample has a total titer 7.48 × 10^13^ vp/mL.

It is important that viral vectors such as rAAV5 are stable, to withstand stress factors and retain their functionality. Maintaining structural integrity is important as the viral vector protects the protein genome, as well as binds to the cell surface for cellular uptake and genome release. Structural change appears to be required for capsid uncoating and genome release. Figure 5A and Figure 7 showed the results of thermal ramps using the Zetasizer Ultra. Between 25 °C and 35 °C, no change in size was observed for full rAAVs (Figure 5A). From 35 °C, the pretransition stage, an increase in size was recorded as highlighted suggesting sample change. The comparison of the intensity particle size distributions at 30 °C and 45 °C (Figure 5B) clearly showed the difference between these samples at these two temperatures.

Figure 9 showed the MADLS size distributions for full and empty rAAV5 capsids measured at neat concentration. Table 6 summarized the hydrodynamic diameters and percentage polydispersity values obtained for the repeat measurements. The percentage polydispersity is a useful parameter for assessing the width of peaks contained in a particle size distribution. It was calculated from Equation (4), where the peak standard deviation is displayed in ZS Xplorer software as the peak width. This was calculated from Equation (5), where X_i_ is the center of each size class of the peak and Y_i_ is the percentage of particles in each size class, according to the intensity of scattered.

% polydispersity = (Peak Standard Deviation/Peak Mean) × 100.
(4)

Peak Standard Deviation = √ ((∑X_i_^2^Y_i_/% Area) − Peak Mean^2^). (5)

Percentage polydispersity is often used in protein research [49] as a predictor of the possible formation of protein crystals [50]. Proteins with low percentage polydispersity values (less than 20%) have good homogeneity, which should result in crystal growth [50].

In the data summarized in Table 6, the empty rAAV5 capsids show significantly larger hydrodynamic sizes and percentage polydispersity values, which could be attributed to the presence of oligomers or, alternatively, a higher capsid flexibility.

The scattering intensity of empty and full AAV5 samples in the range between 50 °C to 70 °C is markedly different (Figure 7). While scattering intensity increased with temperature for the empty AAV5 sample, suggesting aggregation (as evidenced by the increase in hydrodynamic sizes (Figure 5A), the trend was opposite for the full capsid sample. To test if this difference was due to structural change, a thermal ramp study for full rAAV5 capsids was done using different buffer and experimental conditions (Figure 8). The results show that capsid disassembly and structural change could be observed for a range of titers and was affected by the ramp rate and buffer conditions (sample 2 and 3).

The results are encouraging, as they suggest the kinetic-controlled ejection of ssDNA in response to change in capsid structure and stability due to thermal stress. Genome release in response to thermal stress followed by capsid disassembly at higher temperatures was also shown by qPCR, atomic force microscopy, and transmission electron microscopy data, which also linked this to infectivity of the vectors [50,51]. It is worth noting that, as the process seems to be kinetically controlled, the use of a label-free approach to its characterization is beneficial. An extrinsic dye could affect capsid integrity and kinetics of capsid uncoating.

The DLS size results in Figure 5A,B showed an increase in the size of the full capsids with temperature. This would be expected to show an increase in the intensity of scattered light, which was observed for the empty rAAV5 capsids (Figure 7). However, the scattering intensity decreased with increasing temperature for the full rAAV5 capsids (Figure 7). The ELS results summarized in Table 7 provide further information to explain these temperature dependent observations. The more negative zeta potential values obtained for the full rAAV5 capsids (−17.7 mV (±1.9 mV) compared to the empty (−7.7 mV (±2.2 mV)) suggest that the encapsulated ssDNA was released from the full capsids and was located at the capsid surface, as reported by other researchers [50]. This hypothesis would also explain the decrease in scattering intensity seen as a function of temperature. The intensity of scattered light produced by a sample is dependent upon the particle size, the particle concentration, and the optical properties of the particles. As the particle size increased and the concentration was unchanged, the only explanation for the change in scattering intensity of the full capsids observed in this study is a change in the optical properties. The release of encapsulated ssDNA would result in a change in the relative refractive index (the difference in refractive index between the capsid and the dispersant) influencing the scattering capability of the capsids.

Viral capsid stability and function is a balancing act. Viral capsids must be stable enough to contain and protect genome, bind to the host cell surface for cellular uptake, and navigate the cellular milieu, yet they have to offer enough conformational lability to release genome cargo at a replication site.

The mechanism of AAV vector uncoating remains poorly understood. Structural change appears to be required for capsid uncoating and genome release. Based on the published data on thermal stability of AAVs collected with differential scanning fluorimetry [52] and DSC [53], T_m_ of AAV thermal transition is related to capsid disassembly process and serves as indicator of AAV serotype; T_m_ values are generally quite similar for empty and full AAV capsids of one serotype, and they have no clear correlation with capsid dynamics, capsid uncoating, and genome release.

The overlay of the DSC traces recorded in this study for the full and empty rAAV5 samples (Figure 12) provides a way of mapping the thermal stability of rAAV5 beyond T_m_ and correlating it with the thermal trends observed with orthogonal DLS assay.

On the basis of the differences between thermal stability parameters such as T_onset_ and T_m2_ (Table 8) and overall DSC thermogram shape for the empty and full rAAV5 samples, four distinct regions (Figure 12) can be identified on the DSC traces and, considering the findings from DLS thermal ramp experiments, they can be tentatively attributed to the following:

(I) The temperature-induced process observed from about 50 °C only in full rAAV5 sample and extending up to 75 °C for around 30 min. This can be tentatively attributed to the kinetically controlled ejection of ssDNA in response to changes in capsid structure and stability due to thermal stress.
(II) The pretransition process, which was most pronounced in the empty rAAV5 sample.
(III) The main transition, whereby cooperative rAAV5 capsid disassembly was characterized by the T_m_ value specific to AAV5 serotype.
(IV) The additional transition only detected for the full AAV5 sample and tentatively attributed to melting of ssDNA.

## 5. Conclusions

In this study, the capabilities of DLS, MADLS, and SEC–MALS for the analyses of rAAV5 samples in a broad range of viral concentrations (titers) and at different levels of genome loading and sample heterogeneity were evaluated. Linear ranges for rAAV5 size and titer determination were established and reported along with %CV. In addition, the applicability and value of orthogonal and complementary techniques, which are label-free assays not requiring AAV calibration standards, provide information on the following important analytical and quality attributes of viral vectors:

- Capsid size and titer;
- Full/empty ratio and genome titer;
- Aggregate content;
- Fragment content;
- Thermal stability;
- Charge;
- Capsid uncoating.

The SEC–MALS method described in this manuscript is a first-principles technique. It relies on the use of multiple detectors in one and the same run and does not require the use of a combination of separate methods to report AAV viral genome titer, capsid titer, and empty/full ratio. AAV samples are analyzed in their native state. MADLS provides a simple and rapid way to measure capsid titer. Unlike the UV-based methods [54], it does not report the empty/full ratio or genome titer. However, it uniquely complements the measurement of the capsid titer with size, homogeneity, and aggregation levels of AAV samples. Both MADLS and SEC–MALS have an advantage of not being affected by the presence of a significant level of protein or nucleic acid impurities or of buffers components that absorb in the UV range [55].

These label-free biophysical techniques require minimal assay development and can be applied from the capsid design stage through development to formulation development and extended characterization of the drug substance and drug product, strengthening the analytical workflow of in vivo gene therapy development.

## Figures and Tables

**Table 1:** Summary of the critical quality attributes (CQAs) important in viral vector research and the techniques that can provide this information.

Sample Attributes	Relevant Techniques
Capsid size	DLS, SEC–MALS, NTA
Capsid titer or particle count	MADLS, SEC, NTA
Percentage of genome-containing virus particles/% full analysis	SEC–MALS
Aggregate formation	DLS, MADLS, SEC–MALS, NTA
Fragmentation	SEC–MALS
Thermal stability	DLS, DSC
Higher-order structure analysis	DSC
Serotype identification	DSC
Capsid uncoating and genome ejection	DLS, DSC
Binding to receptor	ITC
Charge	ELS

**Table 2:** Quantitative parameters for rAAV5 (Empty).

Parameter	Monomer	Dimer	Aggregates	Fragments
Mw (g/mol)	3.84 × 10^6^	6.98 × 10^6^	1.77 × 10^7^	821,849
Mw/Mn	1.001	1.010	1.175	1.695
Fraction of sample (%)	84.7	7.2	2.7	5.4
Fraction of protein (%)	99.8	-	-	-
Total titer for sample (monomer and aggregate) = 5.91 × 10^13^ vp/mL (2.6% RSD)				

**Table 3:** Quantitative parameters for rAAV5 (Full).

Parameter	Monomer	Aggregates
Mw (g/mol)	4.49 × 10^6^	9.74 × 10^7^
Mw/Mn	1.00	1.22
Fraction of sample (%)	93.2	7.0
Fraction of protein (%)	86.0	-
Mw of protein (g/mol)	3.89 × 10^6^	
% Full rAAV5	78.1	-
Total titer for sample (monomer and aggregate) = 7.48 × 10^13^ vp/mL (3.9% RSD)		

**Table 4:** Full–empty ratios with expected and calculated percentage of full AAV. * As previously calculated starting materials.

Filled–Empty Ratio	% Full rAAV5 Expected	% Full rAAV5 Calculated
4:0	78.1 *	78.1
3:1	58.6	64.5
2:2	39.1	43.6
1:3	19.5	24.3
0:4	0.2 *	0.2

**Table 5:** Effect of thermal stress on monomer fraction and percentage of full rAAV5 sample determined by SEC–MALS.

Stress Time (min)	Monomer Complex Mw (g/mol)	Monomer % Full AAV	Fraction of Monomer
2	4.42 × 10^6^	80	90.3
5	4.47 × 10^6^	79	80.6
10	4.48 × 10^6^	78	78.0
15	4.49 × 10^6^	81	77.5

**Table 6:** Summary of the DLS results for neat concentrations of empty and full rAAV samples measured using backscatter detection.

Measurement	Hydrodynamic Diameter (nm)		% Polydispersity	
	Empty rAAV5	Full rAAV5	Empty rAAV5	Full rAAV5
1	32.8	26.5	33.8	18.2
2	32.2	26.6	34.2	19.5
3	32.1	27.3	35.4	21.1
4	32.5	26.5	34.7	20.5
5	33.1	26.8	33.0	20.5
Mean	32.5	26.7	34.2	18.0
Standard Deviation	0.42	0.34	0.91	1.31

**Table 7:** Electrophoretic mobility and zeta potential values for full and empty rAAV5 samples measured in PBS with 0.001% Pluronic F-68 buffer.

Measurement	Electrophoretic Mobility (m^2^/V·s × 10^−8^)		Zeta Potential (mV)	
	Empty rAAV5	Full rAAV5	Empty rAAV5	Full rAAV5
1	−0.646	−1.047	−9.1	−14.8
2	−0.367	−1.103	−5.2	−15.6
3	−0.561	−1.367	−7.9	−19.3
4	−0.418	−1.311	−5.9	−18.4
5	−0.740	−1.230	−10.4	−17.4
Mean	−0.55	−1.21	−7.7	−17.7
Standard Deviation	0.2	0.1	2.2	1.9

**Table 8:** Thermal stability results of empty and full rAAV5 samples obtained from DSC.

Sample rAAV5	T_onset_, °C	T_m₁_, °C	T_mHalf₁_, °C	T_m₂_, °C	*H*_main tr_, kJ/mol
Empty	78.59	89.43	2.15		2360
Full	65.67	89.68	2.06	94.72	2260

## Data Availability

The data presented in this study are available on request from the corresponding author. The data are not publicly available due to company confidentiality.
